# ECOA-10. Integrated genomic and clinical analysis of BRAF-mutated glioma in adults

**DOI:** 10.1093/noajnl/vdab070.010

**Published:** 2021-07-05

**Authors:** Pinky Langat, Taibo Li, Wenya Linda Bi, Karisa C Schreck

**Affiliations:** 1 Department of Neurosurgery, Brigham and Women’s Hospital, Harvard Medical School, Boston, MA, USA; 2 Center for Neuro-Oncology, Dana-Farber Cancer Institute, Boston, MA, USA; 3 Department of Neuro-Oncology, Johns Hopkins Medical School, Baltimore, MD, USA

## Abstract

*BRAF* alterations are recognized as a significant driver of disease in pediatric low-grade glioma (pLGG) but the implications of *BRAF* alterations on the natural history and response to treatment are unclear in adult glioma. We characterized the molecular and clinical features of a multi-institutional cohort of adults with *BRAF*-mutated gliomas. We identified patients with glioma containing *BRAF* alterations on sequencing in multi-institutional cohorts (Dana-Farber/Brigham Cancer Center, Johns Hopkins Hospital, GENIE, TCGA). *BRAF* alterations were grouped into previously defined classes: I (V600E; RAS-independent/dimerization-independent), II (RAS-independent/dimerization-dependent), III (RAS-dependent/dimerization-dependent) in addition to *BRAF* copy number gains, fusions, and other. We interrogated 289 *BRAF*-altered gliomas (199 patients >=18yrs, 90 patients <18yrs; range 0-85yrs), and observed histopathologic and molecular differences between *BRAF*-altered gliomas in adults versus pediatric patients. Amongst adults, the most common *BRAF* alterations were Class I followed by copy number gains, with glioblastoma (GBM) the most prevalent histology. In comparison, pediatric gliomas in our cohort frequently harbored Class I mutations followed by *BRAF* fusions, with primarily pilocytic astrocytoma and pLGG histologies. Principal component analysis and correlation analysis revealed molecular features associated with gliomas of different *BRAF* alterations and histologies, including mutation of *NF1*, a negative regulator of RAS, which was significantly associated with class II/III *BRAF* alterations (64.3%) and not observed in *BRAF*^*V600E*^-mutated gliomas (0%, n=62) (p<0.0001). Demographic and molecular features were evaluated for correlates for adult glioma risk stratification. Comparative survival analysis showed no significant difference between adult GBM harboring Class I compared to other *BRAF* alterations, whereas young adult age (18-35yrs) was associated with improved outcomes (p<0.05). Among 86 GBM patients with detailed clinicopathologic data, 7 received RAF-targeted therapy, with variable clinical response. This cohort of *BRAF*-altered adult gliomas demonstrates a broad range of molecular alterations with implications for treatment sensitivity and patient risk stratification.

